# Significance of subepithelial deposits in patients diagnosed with IgA nephropathy

**DOI:** 10.1371/journal.pone.0211812

**Published:** 2019-02-20

**Authors:** Mineaki Kitamura, Yoko Obata, Yuki Ota, Kumiko Muta, Hiroshi Yamashita, Takashi Harada, Hiroshi Mukae, Tomoya Nishino

**Affiliations:** 1 Division of Blood Purification, Nagasaki University Hospital, Nagasaki, Japan; 2 Department of Nephrology, Nagasaki University Hospital, Nagasaki, Japan; 3 Department of Nephrology, Nagasaki Renal Center, Nagasaki, Japan; 4 Department of Respiratory Medicine, Unit of Basic Medical Sciences, Nagasaki University Graduate School of Biomedical Sciences, Nagasaki, Japan; Tokushima University Graduate School, JAPAN

## Abstract

Subepithelial deposits are observed in rare adult IgA nephropathy (IgAN) cases and are a key diagnostic finding in IgA-dominant infection-related glomerulonephritis (IgA-IRGN). Sometimes, it is difficult to distinguish IgA-IRGN from IgAN without a precise clinical history. We hypothesized that some IgA-IRGN cases might be diagnosed as IgAN with subepithelial deposits (IgAN-SD) and aimed to clarify the significance of subepithelial deposits in patients diagnosed with IgAN. We examined 464 patients diagnosed with IgAN at Nagasaki University Hospital and affiliated hospitals between 1996 and 2013. The differences in clinicopathological findings between IgAN-SD and IgAN with no subepithelial deposits (IgAN-NSD) were investigated. In addition to clinical data and typical IgAN pathological features, we analyzed complement levels, immunoglobulin localization, light chain staining patterns, and intramembranous deposits. There were 214 men and 250 women with a mean age of 38.8 ± 18.3 years. Subepithelial deposition was observed in 51 patients (11%). Compared to patients with IgAN-NSD, those with IgAN-SD had significantly lower mean serum protein (6.4 g/dL *vs*. 6.7 g/dL; p = 0.02), albumin (3.7 g/dL *vs*. 3.9 g/dL; p = 0.02), and complement (C3) (94 mg/dL *vs*. 103 mg/dL; p = 0.02) levels. Diffuse mesangial hypercellularity (M) (65% *vs*. 45%; p<0.01), endocapillary hypercellularity: (E) (43% *vs*. 28%; p = 0.03), and IgA staining in the glomerular capillary wall (22% *vs*. 8%; p<0.01) were more common in patients with IgAN-SD. The incidence of light chain lambda predominance was lower in patients with IgAN-SD (47% *vs*. 63%; p = 0.03). Hump-shaped subepithelial deposits and intramembranous deposits were observed in nine and 17 patients with IgAN-SD, respectively. Patients with IgAN-SD tended to have the characteristics of IgA-IRGN rather than IgAN-NSD. Since the therapeutic strategies for IgA-IRGN differ from those for IgAN, we should review the clinical history and pay careful attention to the clinical course in cases with atypical findings, such as subepithelial deposits.

## Introduction

IgA nephropathy (IgAN) is defined by the presence of dominant or co-dominant mesangial IgA immune deposits, often accompanied by C3 deposits and abnormal findings on urinalysis, and the exclusion of other etiologies for IgA deposition [1,2]. The light microscopic features of IgAN are diverse; mesangial lesions vary from minimally changed to diffusely proliferated, and may have sclerosis and/or crescents [3]. Although electron dense deposits (EDDs) in the mesangial area are one of the typical electron microscopic findings of IgAN, some cases show additional deposits involving the glomerular capillary wall [4,5]. The clinicopathological features of IgAN differ among patients; hence, several factors are thought to be associated with the onset and progression of IgAN [2,6,7].

About 30 years ago, a few studies investigated the location of EDDs in IgAN and speculated that capillary wall deposits, including subepithelial deposits, were associated with its severity [4,5]. Several cases and case-series of IgA-dominant infection-related glomerulonephritis (IgA-IRGN), such as methicillin resistant staphylococcus aureus (MRSA)-related glomerulonephritis, have been reported since 1995 [8–11]. The clinical features of IgA-IRGN resemble those of post-streptococcal infection-related glomerulonephritis in terms of endocapillary hypercellularity, hypocomplementemia, and hump-shaped subepithelial deposits [8,11]. During the course of healing, the subepithelial deposits shrink and move to the intramembranous area [11]. Therefore, past studies have noted a difficulty in distinguishing IgA-IRGN from IgAN without a precise clinical history [10–13].

Although previous studies had shown that the subepithelial deposits in IgAN were associated with the severity of urinary abnormality, especially proteinuria [4], the clinicopathological significance of subepithelial deposits remains unknown. Some reports have proposed that several findings are useful for distinguishing IgA-IRGN from IgAN, such as subepithelial deposits, hypocomplementemia, and the absence of lambda predominance [11,12]. However, few studies have validated these findings in patients diagnosed with IgAN.

In this study, we investigated IgAN patients while focusing on subepithelial deposit-positive patients diagnosed as having “IgA nephropathy” and hypothesized that some patients with IgAN with subepithelial deposits (IgAN-SD) might have been diagnosed with IgA-IRGN because of the lack of appropriate clinical histories. To elucidate the clinicopathological characteristics of IgAN-SD, we compared patients with IgAN-SD and those with IgAN with no subepithelial deposits (IgAN-NSD).

## Materials and methods

This was a cross sectional study. We examined patients diagnosed with IgAN at Nagasaki University Hospitals and its affiliated hospitals between 1996 and 2013. The exclusion criteria for this study were age <15 years at renal biopsy; incomplete immunofluorescence data; and the presence of secondary causes of mesangial IgA deposits, such as IgA vasculitis, lupus erythematosus, IgA-IRGN, and IgAN complicated with membranous nephropathy. Patients in whom the glomerulus could not be observed by electron microscopy were also excluded.

We collected the demographic data of patients, including age, sex, blood examinations, and urinary tests, from their renal biopsy request forms. We also collected pathological data, including light microscopy, immunofluorescence staining, and electron microscopy data. As the standard of hypocomplementemia; we adopted the following standard, CH50; <30 U/mL, C3;<73 mg/dL, and C4<11 mg/dL.

Light microscopic analysis was performed based on the 2016 revision of the Oxford classification [14]. In brief, the following were assessed: mesangial cellularity score, <0.5 (M0) or >0.5 (M1); endocapillary hypercellularity, absent (E0) or present (E1); segmental glomerulosclerosis, absent (S0) or present (S1); interstitial fibrosis/tubular atrophy, ≤25% (T0), 26–50% (T1), or >50% (T2); and cellular/fibrocellular crescents, absent (C0), present in at least 1 glomerulus (C1), or present in >25% of glomeruli (C2).

The immunofluorescence studies were performed using direct fluorescent antibodies to human IgG, IgA, IgM, C3, C1q, fibrinogen, kappa, and lambda. The amount and extent of fluorescence were graded from 0 to 4+, and positive cases were defined as those ≥1+. The light chain staining pattern was classified into two groups: lambda-dominant group and non-lambda-dominant group, which included kappa-dominant cases and lambda and kappa-equivalent cases [15].

Electron microscopic findings were evaluated for the presence of subepithelial and endothelial deposits and mesangial interposition. In IgAN-SD, the characteristics of EDDs were also evaluated; for example, hump-shape and the presence or absence of accompanying intramembranous deposits.

For comparing the characteristics of IgA-IRGN and IgAN-SD, we also collected the information of cases diagnosed as IgA-IRGN in our hospital.

Clinicopathological findings were compared between patients with IgAN-SD and those with IgAN-NSD. Statistical analyses were performed using the Wilcoxon rank-sum test for continuous variables and the chi-squared test for categorical variables using JMP 13 software (SAS Institute Inc., Cary, NC). The missing data were removed from the analyses and only the remaining data were used.

This study was approved by the ethics committee of Nagasaki University Hospital (Nagasaki, Japan) (18082002). Although all patients in this study were informed, since this was a cross-sectional study, the ethics committee waived the need for informed consent.

## Results

The number of eligible patients was 464. There were 214 men and 250 women with an overall mean age of 38 ± 18 years. The demographic data and pathological findings of all patients are shown in Table 1. There were 51 patients with subepithelial deposits (IgAN-SD group) and 413 without (IgAN-NSD group) (Table 1).

**Table 1:** The demographic and clinicopathological characteristics of the patients and the differences in clinicopathological and demographic characteristics between IgAN-SD and IgAN-NSD patients.

	Total	IgAN-SD	IgAN-NSD	p-value
**Numbers**	464	51	413	
**Age (years)**	38.8 ± 18.3	39.3 ± 19.7	38.8 ± 18.2	0.99
**Sex**	M, 214; F, 250	M, 25; F, 26	M, 189; F, 224	0.66
**Height (cm)**	162 ± 9	161 ± 10	162 ± 9	0.33
**Body weight (kg)**	59.1 ± 12.2	57.7 ± 11.2	59.3 ± 12.4	0.46
**sBP (mmHg)**	125 ± 19	125 ± 18	125 ± 19	0.61
**dBP (mmHg)**	73 ± 13	74 ± 12	73 ± 13	0.53
**Hb (g/dL)**	13.0 ± 1.9	12.9 ± 1.8	13.0 ± 1.9	0.71
**WBC (/μL)**	6506 ± 1846	6669 ± 1753	6486 ± 1858	0.35
**CRP (mg/dL)**	0.46 ± 1.47	0.53 ± 1.32	0.45 ± 1.49	0.71
**TP (g/dL)**	6.7 ± 0.8	6.4 ± 0.9	6.7 ± 0.8	0.02
**Alb (g/dL)**	3.9 ± 0.7	3.7 ± 0.8	3.9 ± 0.7	0.02
**T-Chol (mg/dL)**	201 ± 53	204 ± 63	200 ± 51	0.73
**TG (mg/dL)**	131 ± 109	115 ± 82	134 ± 112	0.42
**BUN (mg/dL)**	16.1 ± 8.6	16.6 ± 11.4	16.1 ± 8.2	0.92
**Cr (mg/dL)**	0.98 ± 0.83	0.99 ± 0.81	0.98 ± 0.83	0.99
**UA (mg/dL)**	5.7 ± 1.8	5.8 ± 1.6	5.7 ± 1.8	0.46
**IgA (mg/dL)**	351 ± 152	343 ± 152	352 ± 152	0.70
**CH50 (U/mL)**	41 ± 9	39 ± 10	42 ± 9	0.14
**C3 (mg/dL)**	102 ± 23	94 ± 26	103 ± 23	0.02
**C4 (mg/dL)**	24 ± 8	24 ± 7	25 ± 9	0.89
**U-Pro (g/gCr)**	1.50 ± 2.49	2.02 ± 1.99	1.42 ± 2.55	0.01
**U-abnormality (months)**	56 ± 90	63 ± 108	56 ± 87	0.57
**Hypocomplementemia**	11%	12%	10%	0.72
**Nephrotic syndrome**	5.2%	7.8%	4.8%	0.36
**Hematuria**	**Number (%)**	**Number (%)**	**Number (%)**	
0–4/HPF	83 (18%)	3 (6%)	80 (19%)	
5–9/HPF	53 (11%)	5 (10%)	48 (12%)	
10–19/HPF	72 (16%)	9 (18%)	63 (15%)	
20–49/HPF	113 (24%)	15 (29%)	98 (24%)	
50–99/HPF	57 (12%)	5 (10%)	52 (13%)	
>100/HPF	80 (17%)	14 (27%)	66 (16%)	
History of macrohematuria	86 (19%)	12 (24%)	74 (18%)	0.33
**Oxford Classification**	**Number (%)**	**Number (%)**	**Number (%)**	
M0	245 (53%)	18 (35%)	227 (55%)	
M1	219 (47%)	33 (65%)	186 (45%)	0.008
E0	325 (70%)	29 (57%)	296 (72%)	
E1	139 (30%)	22 (43%)	117 (28%)	0.03
S0	292 (63%)	34 (67%)	258 (63%)	
S1	172 (37%)	17 (33%)	155 (37%)	0.56
T0	323 (70%)	35 (69%)	288 (70%)	
T1	127 (27%)	14 (27%)	113 (27%)	
T2	14 (3%)	2 (4%)	12 (3%)	0.92
C0	272 (59%)	27 (53%)	245 (59%)	
C1	154 (33%)	19 (37%)	135 (33%)	
C2	38 (8%)	5 (10%)	33 (8%)	0.68
**Light Microscopy**	**Mean ± SD**	**Mean ± SD**	**Mean ± SD**	
Total glomeruli	20.2 ± 12.3	18.8 ± 9.9	20.4 ± 12.6	0.26
Crescent (%)	8.7 ± 15.2	7.0 ± 10.0	8.9 ± 15.7	0.63
Global Sclerosis (%)	14.3 ± 18.5	13.7 ± 19.1	14.6 ± 18.6	0.50
**Immunofluorescence**	**Number (%)**	**Number (%)**	**Number (%)**	
IgG-M	74 (16%)	10 (20%)	64 (16%)	0.38
IgG-GBM	7 (2%)	2 (4%)	5 (1%)	0.13
IgA-M	464 (100%)	51 (100%)	413 (100%)	
IgA-GBM	45 (10%)	11 (22%)	34 (8%)	0.002
IgM-M	325 (70%)	36 (70%)	289 (70%)	0.93
IgM-GBM	17 (4%)	4 (8%)	13 (3%)	0.13
C3-M	423 (91%)	47 (92%)	376 (91%)	0.83
C3-GBM	29 (6%)	10 (20%)	19 (5%)	<0.001
C1q-M	40 (9%)	4 (8%)	36 (9%)	0.83
C1q-GBM	3 (1%)	0 (0%)	3 (1%)	0.54
lambda-dominant	284 (61%)	24 (47%)	260 (63%)	0.03
**Electron microscopy**	**Number (%)**	**Number (%)**	**Number (%)**	
Subendothelial deposits	58 (13%)	11 (22%)	47 (11%)	0.045
Mesangial interposition	214 (47%)	34 (67%)	180 (44%)	0.003

SD: standard deviation, IgAN-SD: IgA nephropathy with subepithelial deposits, IgAN-NSD: IgA nephropathy with subepithelial deposits, Ig: immunoglobulin, sBP: systolic blood pressure, dBP: diastolic blood pressure, Hb: hemoglobin, WBC: white blood cell, CRP: C reactive protein, TP: total protein, Alb: albumin, T-Chol: total cholesterol, TG: triglyceride, BUN: blood urea nitrogen, Cr: creatinine, UA: uric acid, U-abnormality: duration of urinary-abnormality, HPF: high power field, GBM: glomerular basement membrane.

**Fig 1:**
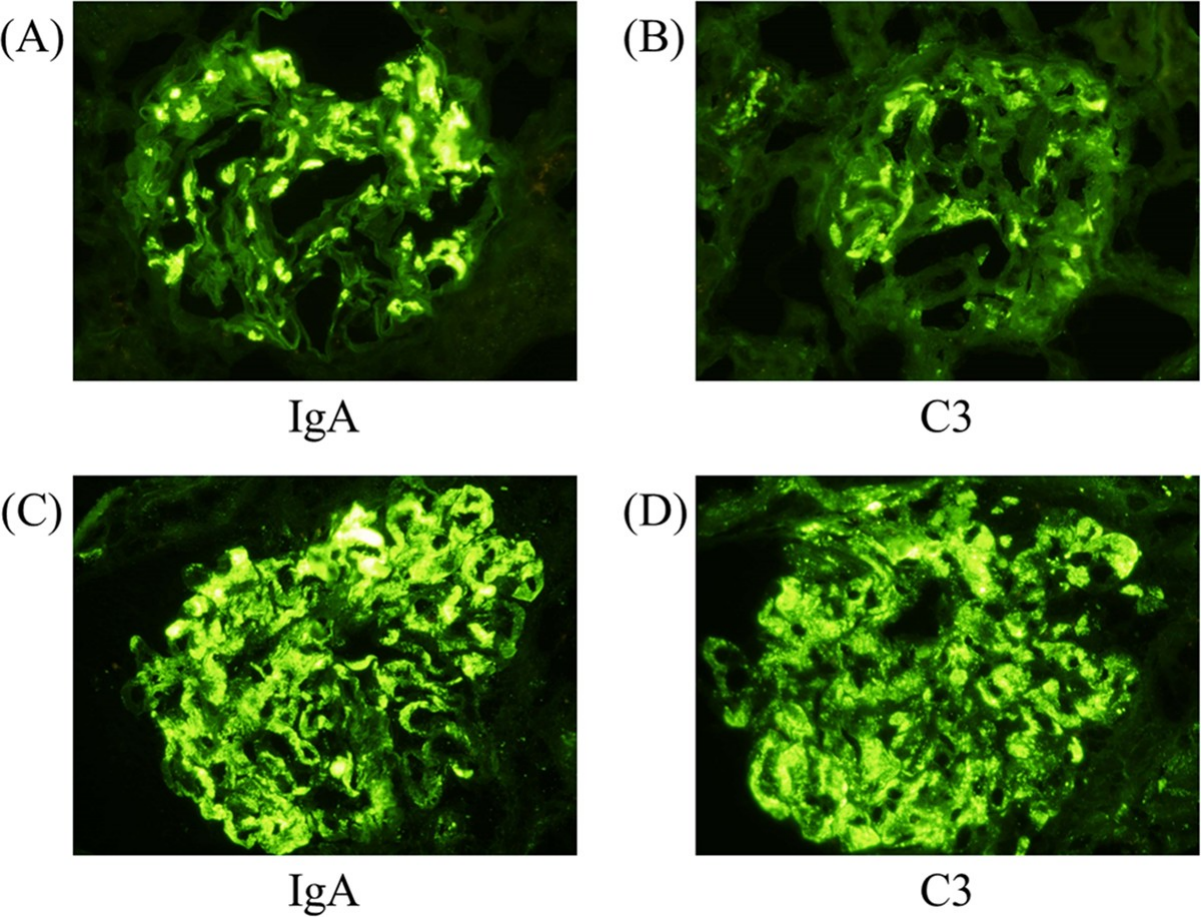
Representative immunofluorescence patterns of IgA and C3. (A) IgA deposition and (B) C3 deposition in mesangial areas in a typical IgA nephropathy case. (C) IgA deposition and (D) C3 deposition in both mesangial and capillary areas in an atypical IgA nephropathy case. All magnifications of photos are x400.

Compared to patients with IgAN-NSD, those with IgAN-SD showed higher levels of proteinuria, lower proteinemia, and lower serum C3. The mesangial cellularity score (M), incidence of endocapillary hypercellularity (E), and IgA staining in the glomerular capillary walls were higher in the IgAN-SD group. The representative figures of IgA and C3 showing mesangial and/or capillary deposition are shown in Fig 1.

The light chain staining patterns were lambda-dominant in 284 patients, lambda and kappa-equivalent in 159, and kappa-dominant in 21. Therefore, there were 284 patients with lambda-dominance and 180 without. The incidence of light chain lambda-predominance was lower in patients with IgAN-SD. Subendothelial deposits and mesangial interposition tended to be seen in patients with IgAN-SD (Table 1). Hump-shaped subepithelial deposits were observed in nine patients (18%) and intramembranous deposits in 17 (33%).

Among the patients with hump-shaped subepithelial deposits, four presented with lower serum C3 levels, three showed endocapillary hypercellularity, and the intensity of immunofluorescence of C3 was codominant or more dominant than that of IgA in six patients. Case 4, 5, and 8 had hypercellularity and neutrophils were observed in these three cases in hypercellularity lesions, however, the numbers of neutrophils were quite a few.

The period of this study was about 18 years; we divided the period into the earlier period (1996–2004) and the later period (2005–2013) and compared the prevalence of IgAN-SD between the two periods. The proportion of IgAN-SD was 13.0% in the earlier period and 9.8% in the later period, with no significant difference (p = 0.29)

**Fig 2:**
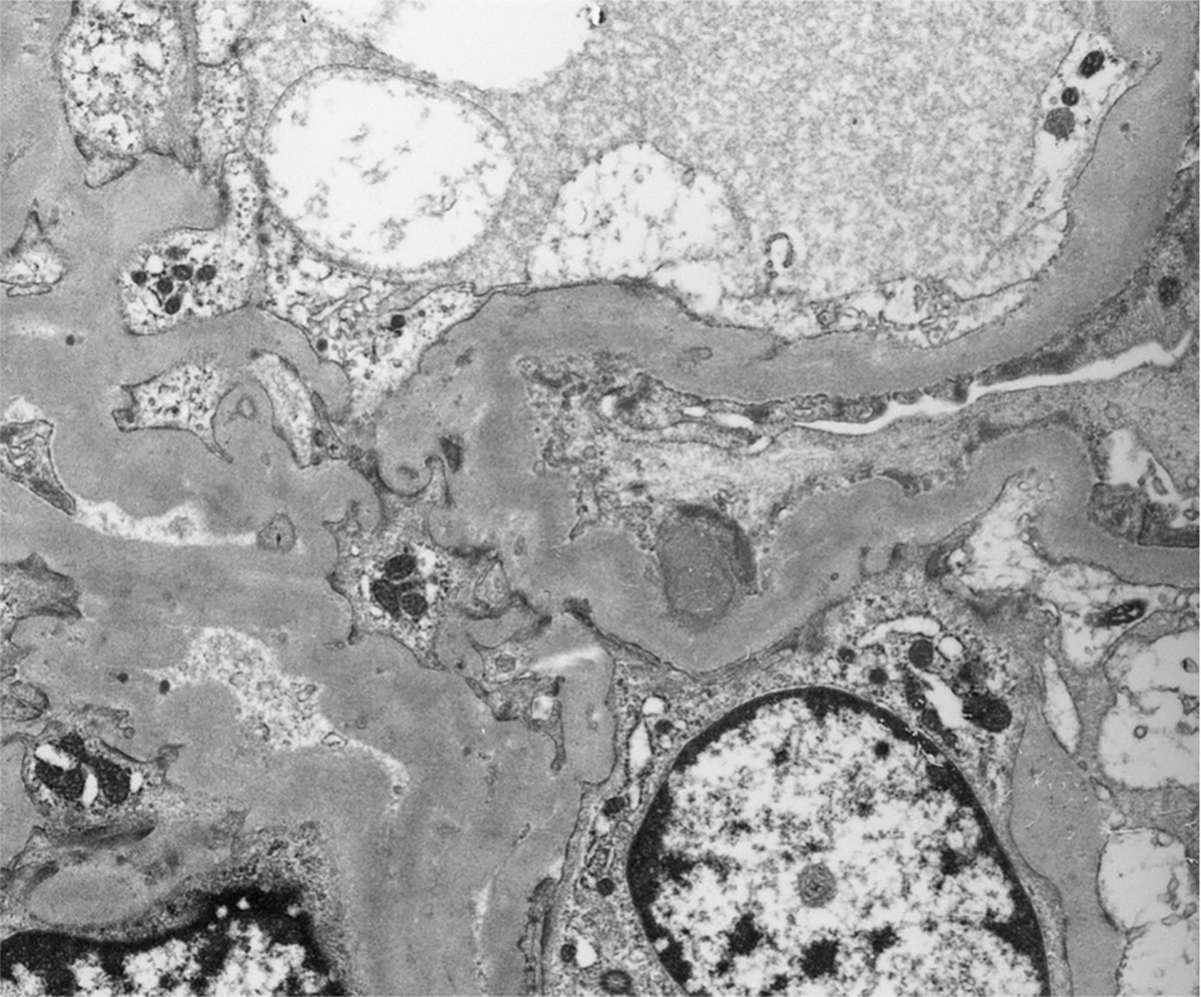
Electron microscopic photograph of representative case of hump-shaped subepithelial deposits in IgAN-SD. Case 7 in Table 2 upper side is shown.

The clinicopathological features of patients with hump-like subepithelial deposits and diagnosed IgA-IRGN in our hospital are summarized in Table 2 and the electron microscopic photo of a representative case is shown in Fig 2.

**Table 2:** Clinical characteristics of IgAN-SD patients with hump-like subepithelial deposits and diagnosed IgA-IRGN cases.

	Age	Sex	DM	Pre infect.	Cr (mg/dL)	C3 (mg/dL)	C4 (mg/dL)	U-pro (g/gCr)	U-RBC /HPF	Oxford classification					Neu	IgA M	IgA GBM	C3 M	C3 GBM	λ-D	EDD locat.
										M	E	S	T	C							
IgAN-SD with hump-shaped subepithelial deposits																					
1	18	M	(-)	(+)	0.8	88	18	1.8	>100	0	0	0	1	1	(-)	3+	1+	2+	1+	No	MEpEn
2	22	M	(-)	(+)	0.7	34	38	0.1	>100	1	0	0	0	0	(-)	1+	-	1+	1+	No	MEp
3	23	M	(-)	(-)	0.7	112	31	0.2	50–99	1	0	0	0	1	(+)	2+	-	1+	-	Yes	MEp
4	38	F	(-)	(-)	1.1	68	24	2.6	20–29	0	1	0	0	1	(+)	1+	-	2+	1+	Yes	MEp
5	39	F	(-)	(-)	0.7	41	16	1+*^1^	15–20	1	1	0	0	0	(+)	1+	-	1+	-	No	MEp
6	51	F	(-)	(-)	0.5	17	22	-*^1^	20–29	1	0	0	1	0	(+)	1+	-	1+	-	Yes	MEp
7	61	M	(+)	(-)	1.1	NA	NA	2.0	>100	1	0	0	1	2	(-)	1+	-	1+	-	No	MEp
8	75	F	(-)	(+)	0.6	81	17	8.3	1–4	1	1	0	0	0	(+)	1+	-	-	-	No	MEpEn
9	80	M	(+)	(-)	5.5	95	22	1.9	>100	1	0	0	1	0	(-)	1+	-	1+	-	Yes	MEp
	Age	Sex	DM	Pre infect.	Cr (mg/dL)	C3 (mg/dL)	C4 (mg/dL)	U-pro (g/gCr)	U-RBC /HPF	Oxford classification					Neu	IgA M	IgA GBM	C3 M	C3 GBM	λ-D	EDD locat.
										M	E	S	T	C							
Cases diagnosed as IgA-IRGN																					
1	18	M	(-)	(+)	0.5	95	22	1.9	>100	1	1	0	0	0	(-)	1+	-	-	-	NA	MEpEn
2	48	M	(-)	(+)	1.4	NA	NA	0.08	5–9	0	0	0	2	0	(-)	1+	-	1+	-	NA	M
3	79	M	(-)	(+)	3	73	21	1+*^1^	>100	0	0	0	0	1	(-)	2+	2+	1+	2+	No	MEpEn
4	56	M	(+)	(+)	0.8	134	35	7.2	>100	0	1	0	0	0	(+)	1+	-	1+	-	NA	M
5	69	M	(-)	(+)	3.7	124	32	3	>100	1	1	0	1	1	(+)	1+	-	1+	-	No	M
6	60	M	(+)	(+)	4.79	115	35	6.4	11–20	0	1	0	0	1	(+)	1+	-	1+	-	NA	MEpEn
7	78	M	(-)	(+)	2.94	64	30	10.1	5–9	1	1	0	1	1	(+)	2+	1+	3+	2+	No	NA

Age: years, IgAN-SD: IgA nephropathy with subepithelial deposits, M: male, F: female, DM: diabetes mellitus, Pre infect.: histories of infection before biopsy written in renal biopsy request forms, Cr: serum creatinine (mg/dL), C3: serum C3 (mg/dL), C4: serum C4(mg/dL), U-Pro: protein-to-creatinine ratio (g/gCr), U-RBC: urinary sediment red blood cells/ high power field, M: Oxford classification M, E: Oxford classification E, S: Oxford classification S, T: Oxford classification T, C: Oxford classification C, Neu: neutrophil infiltration in capillary, IF IgA: intensity of immune fluorescence of IgA, IF C3: intensity of immune fluorescence of C3, λ-D: Lambda dominance in immune fluorescence, EDD locat.: Electron dense deposits location, M:mesangium Ep: subepithelial En: Subendothelial, SEnD: subendothelial deposits, NA: not available

*1 The results of quantitative urinary protein were not available

The diagnosed IgA-IRGN cases were all in men and those who tended to have diabetes; moreover, infiltrating neutrophils were observed in cases with endocapillary hypercellularity lesions. All cases had histories of infection, which were associated with MRSA or methicillin-susceptible Staphylococcus aureus before renal biopsy. However, there were only few cases of IgA-IRGN and statistical analyses were not performed.

## Discussion

In the present study, we investigated 464 cases of IgAN and 51 of IgAN-SD. Our results showed that subepithelial deposits may be associated with lower serum protein levels, lower serum C3 levels, mesangial proliferation, endocapillary hypercellularity, higher incidence of IgA staining in the glomerular capillary wall, and absence of lambda-predominance. Among the patients with IgAN-SD, nine (18%) showed hump-shaped subepithelial deposits.

The clinical significance of subepithelial deposits in IgAN remains unclear; however, previous studies have shown that the prevalence of subepithelial deposit-positive cases is about 13–18% [5,13] and that they seemed to be associated with indicators of clinical severity, such as the extent of proteinuria and renal function [4,5]. Subepithelial deposits tended to be more frequently observed in IgAN patients with mesangial and capillary wall deposits (50%) than in those with only mesangial deposits (3%) [4]. In this study, the incidence of subepithelial deposits was 11%, and patients with IgAN-SD exhibited more severe proteinuria and a higher incidence of capillary wall staining compared to patients with IgAN-NSD. These findings were consistent with previous reports [4,5]. Patients with IgAN-SD also showed lower serum C3 levels and an absence of lambda-dominance compared with those with IgA-NSD. These findings were not mentioned in previous studies [4,5] and are also typical features of infection-related glomerulonephritis, not limited to IgA dominant cases.

No established clinical criteria for the diagnosis of IgA-IRGN have been developed. However, a previous study used the presence of at least three of the following criteria for diagnosis [16]: (1) clinical or laboratory evidence of infection preceding or concurrent with onset, (2) reduced serum complement, (3) endocapillary proliferative and exudative glomerulonephritis, (4) C3-dominant or co-dominant glomerular immunofluorescence staining, and (5) hump-shaped subepithelial deposits. Among these criteria, the clinical history of infection should be the most important. However, the hump-like subepithelial deposits seen in IgAN-SD cases 4 and 5 could be diagnosed as IgA-IRGN using these criteria despite the absence of a history of infection (Table 2).

The interpretation of infectious clinical history and etiology is sometimes difficult in both IgAN and IgA-IRGN. MRSA is the most famous etiology for IgA-IRGN, and patients tend to be elderly and diabetic. However, other pathogens, such as gram-negative rods, are known to cause IgA-IRGN, and in such cases the patients are not limited to diabetics and the elderly [17,18]. On the other hand, various infections, such as tonsillitis, exacerbate IgAN and may be associated with its onset according to the multi-hit theory [19,20]. Although the short latency period and gross hematuria after infection are specific to IgAN, these features are not observed in all cases.

According to previous reports, the ultrastructural features of IgA-IRGN resemble those of poststreptococcal glomerulonephritis. IgA-IRGN has been subclassified into three stages according to the healing process: acute, subacute, and persistent/resolving [11,21]. In the acute stage, diffuse endocapillary hypercellularity with prominent neutrophil infiltration and prominent subepithelial humps exists. In the subacute stage, the glomeruli show mesangial proliferation in addition to segmental endocapillary hypercellularity with few neutrophils and/or subepithelial humps showing evidence of resorption. Loss of neutrophils, mesangial proliferation, and resorbed subepithelial and intramembranous deposits characterize the persistent/resolving stage. Based on these characteristics, IgA-IRGN could be misdiagnosed as IgAN when in the subacute or persistent/resolving stages [11].

The mechanisms underlying subepithelial deposition are thought to be associated with endocapillary hypercellularity, while proteolytic enzyme-producing infiltrating cells and basement membrane injury result in proteinuria [22,23]. In this study, the incidence of endocapillary hypercellularity was significantly higher in patients with IgAN-SD. Therefore, subepithelial deposits could be caused by endocapillary hypercellularity, similarly to infection-related glomerulonephritis.

Previous studies have shown a lambda-dominant mesangial light chain staining pattern in IgAN [15,24,25], although concomitant IgG or IgM staining in the mesangial area may affect the interpretation of light chain staining patterns. Conversely, staining for kappa was equal or stronger in more than two-thirds of patients with IgA-IRGN [11,16]. Although the precise mechanism remains unclear, a previous report showed that the proportion of circulating IgA-kappa/IgA-lambda in patients with IgAN was lower than that in healthy controls [26]. In contrast, patients with IgA-IRGN may not exhibit the decreased kappa/lambda ratio of IgA and would thus have different light chain staining patterns. Therefore, light chain staining patterns could be a candidate tool for distinguishing IgA-IRGN from IgAN.

There are several limitations to this study. First, recent research has shown that immunofluorescence analysis using KM55 monoclonal antibody can clearly distinguish IgAN and IgA vasculitis from other etiologies of IgA deposition [27]. It may be useful to distinguish IgA-IRGN from IgAN. However, we did not perform immunofluorescence analysis using KM55 in this study because the available renal tissues were limited among the included patients. We want to elucidate whether KM 55 positive rate differs between IgAN-SD and IgAN-NSD in a future study. Second, although the clinical history is critical to renal pathological diagnoses, our diagnoses depended on renal biopsy request forms, and some cases lacked a precise clinical history, especially with respect to infection. The history of infection, such as tonsillitis or upper respiratory infections, was written in the renal biopsy request forms of only 90 cases (19%). However, this reporting would be lower than the prevalence of the actual history, because some renal biopsy request forms were poorly described and information bias should have existed. Moreover, the written anti-streptolysin O information was available in 117cases (25%) of the renal biopsy request forms. Third, this study was conducted between 1996 and 2013, while Koyama *et al*. proposed IgA-IRGN in 1995. Therefore, diagnoses early in the study period were made without specific awareness of IgA -IRGN. As shown in the results, the proportion of IgAN-SD was higher in the earlier period than those in the later period; however, there was no significant difference. Fourth, clinical data were lacking in several cases. Despite this, the renal biopsy request forms were almost identical during the study period. Furthermore, the results of blood and urinary examinations may differ among facilities due to varying equipment and measurement methods.

## Conclusions

Obtaining clinical histories is crucial for renal pathological diagnosis and lack thereof may lead to misdiagnosis or diagnosis of the most probable disease. Therefore, patients with IgA-IRGN without a precise infection history may be diagnosed with IgAN. The atypical findings in IgAN might aid in distinguishing it from IgA-IRGN. When encountering patients with the unusual pathological findings of IgAN, such as subepithelial deposits, their medical record or clinical history must be thoroughly reviewed. On the other hand, when encountering the unusual features of IgAN on blood examinations, light microscopy, and immunofluorescence studies, electron microscopy analysis should be performed promptly to validate the treatment policy.

## Supporting information

S1 Data Patients’ data are supplied in a supporting information file. (XLSX)
